# Allogeneic endometrial regenerative cells: An "Off the shelf solution" for critical limb ischemia?

**DOI:** 10.1186/1479-5876-6-45

**Published:** 2008-08-19

**Authors:** Michael P Murphy, Hao Wang, Amit N Patel, Suman Kambhampati, Niren Angle, Kyle Chan, Annette M Marleau, Andrew Pyszniak, Ewa Carrier, Thomas E Ichim, Neil H Riordan

**Affiliations:** 1 Division of Vascular Surgery, Indiana University School of Medicine, Indiana, USA; 2 Department of Surgery, University of Western Ontario, London, Canada; 3 Dept of Cardiothoracic Surgery, University of Utah, Salt Lake City, USA; 4 VA Medical Center, Kansas City, USA; 5 Dept Vascular and Endovascular Surgery, University of California San Deigo, USA; 6 Medistem Inc, San Diego, California, USA; 7 The Scripps Research Institute, San Diego, USA; 8 Orcrist Bio, Calgary, Canada; 9 University of California Cancer Center, San Diego, California, USA

## Abstract

Critical limb ischemia (CLI) is an advanced form of peripheral artery disease which is responsible for approximately 100,000 amputations per year in the US. Trials to date have reported clinical improvement and reduced need for amputation in CLI patients receiving autologous bone marrow or mobilized peripheral blood stem cells for stimulation of angiogenesis. While such treatments are currently entering Phase III trials, practical and scientific pitfalls will limit widespread implementation if efficacy is proven. Hurdles to be overcome include: a) reduced angiogenic potential of autologous cells in aged patients with cardiovascular risk factors; b) invasiveness/adverse effects of bone marrow extraction and G-CSF mobilization, respectively; and c) need for on-site cellular manipulation. The Endometrial Regenerative Cell (ERC) is a mesenchymal-like stem cell derived from the menstrual blood that is believed to be associated with endometrial angiogenesis. We discuss the possibility of using allogeneic ERCs as an "off the shelf" treatment for CLI based on the following properties: a) High levels of growth factors and matrix metalloprotease production; b) Ability to inhibits inflammatory responses and lack of immunogenicity; and c) Expandability to great quantities without loss of differentiation ability or karyotypic abnormalities.

## Critical limb ischemia

Critical limb ischemia (CLI) is classically defined as chronic ischemic rest pain, ulcers, or gangrene due to proven occlusive disease [1]. It is diagnosed based on symptomology such as burning rest pain in the distal foot or lack of foot pulses, as well as using objective measurements including low ankle pressure (< 50–70 mm Hg), reduced toe pressure (< 30–50 mm Hg), reduced transcutaneous oxygen (TPCO2 (< 30–50 mm Hg) and ankle brachial index (ABI) of < 0.6. Occlusion is demonstrated by angiography or duplex ultrasound scanning. CLI is caused by arterial occlusion affecting the limbs, usually caused by atherosclerosis or in a smaller number of patients by thromboangiitis obliterans (Buerger's Disease), or arteritis.

This condition is a major cause of morbidity and mortality: Approximately 20–45% of patients require amputation, and 1-year mortality is estimated to be as high as 45% in patients who have undergone amputation [2]. The quality of life of CLI patients has been compared by some authors to that of terminal cancer patients [3]. Current treatment options for this group of patients are limited. According to the Inter-Society Consensus for the Management of Peripheral Arterial Disease (TASC II) treatment for CLI should be focused on revasularization using surgical or percutanous means [1]. Unfortunately less than half of the patients are eligible for these procedures, and efficacy is limited. Additionally, many patients require additional procedures due to high levels of restenosis. Non-surgical options for CLI are limited to medical therapy, which offers limited or no benefit. The dismal state of options for this patient population was best surmised by Schainfeld and Isner in the statement "Critical limb ischemia: nothing to give at the doctors office" [4]. Given the poor prognosis associated with CLI, numerous interventions have been attempted, primarily based on stimulation of angiogenesis in order to allow formation of collateral blood vessels.

## Cytokine mediated angiogenesis for CLI

Angiogenesis therapy has been described as a "biological bypass", the idea being that through administration of agents capable of inducing collateralization, a more natural type of "bypass" can be achieved. Indeed it has been observed that ischemic muscles secrete angiogenic factors in response to hypoxia and that to some extent natural angiogenesis does occur in animal models of CLI and in humans [5,6]. One of the angiogenic factors noted in many ischemic conditions, including cardiac ischemia, stroke, and CLI is vascular endothelial growth factor (VEGF) [7-9]. In 1994, Isner's group sought to enhance ischemia-associated angiogenesis using single bolus intra-arterial administration of VEGF-165 in a rabbit model of CLI. Rabbits with resected femoral arteries demonstrated augmentation of perfusion, increased capillary density, and overall better function as compared to control rabbits [10]. Subsequent experiments sought to optimize therapeutic effect using different dosing schedules. Daily VEGF-165 administration for 10 days subsequent to ligation and resection of the external iliac artery and femoral artery, respectively was performed [11]. Not only was dose-dependent increase in collateralization observed, but rabbits receiving the highest dose of VEGF-165 (1 mg) had no incidence of calf muscle atrophy and distal limb necrosis, whereas this was present in 85.7% of control rabbits. A similar study in the rabbit model of CLI demonstrated superior benefit in terms of limb function in animals receiving VEGF by osmotic pump for 28-days in comparison to nitroglycerin or saline treatment [12]. A variety of studies have been performed with VEGF protein which confirmed the proangiogenic, as well as angiogenesis-independent muscle reparative properties in the limb ischemia model [13-15]. These positive preclinical data unfortunately were not successfully reproduced in the clinic. Trials using VEGF protein [16], or DNA, did not show significant benefit at reducing leg amputations in a double blind setting [15,17]. One of the concerns with VEGF therapy is its vasodilatory effects, as well as induction of blood vessels that are relatively immature and "leaky".

Another approach involved use of the cytokine fibroblast growth factor-1 (FGF-1). Given that FGF-1 is considered "upstream" of VEGF, it is believed to stimulate numerous angiogenic processes so as to result in creation of more mature vessels [18]. Indeed, it is believed that one of the differences between tumor neo-vasculature, which is characteristically leaky, and neo-vasculature associated with physiological angiogenesis, is the ratio of VEGF to FGF [19]. Specifically, it was demonstrated both in animal models [20] and in clinical trials [21] that muscular ischemia is associated with up-regulation of FGF gene transcription, suggesting that this is part of the endogenous pro-angiogenic response to ischemia. The critical role of FGF in endogenous angiogenesis was conclusively demonstrated in FGF knockout mice, which displayed inhibited ability to heal post-wounding [22]. Although FGF-1 gene therapy has clinically been used in CLI patients with some improvement in ABI and perfusion, statistically powered randomized clinical trials have not been conducted to date [23].

It is known that additive and/or synergistic effects are observed in terms of neoangiogenesis when several angiogenic factors are combined. For example, Cao et al demonstrated synergy between administration of PDGF-BB and FGF-2 in terms of increasing blood vessel formation and function in the femoral artery ligation model in rats and rabbits [24]. Similarly, in cancer angiogenesis, it is known that several tumor-derived angiogenic factors synergize for acceleration of neovascularization [25]. Accordingly, investigators have attempted to activate upstream mediators of several angiogenic signals through transfection of genes encoding transcription factors such as HIF-1 alpha [26]. In fact, this approach has been demonstrated to be superior to VEGF gene administration in terms of new capillary sprouting. In a Phase I dose-escalating trial, transfection of HIF-1 alpha into CLI patients demonstrated tolerability with some indication of efficacy [27].

In conclusion, while administration of angiogenic factors to patients with CLI does induce some benefit in early trials, data from randomized trials to date do not support widespread use. The transfection of upstream transcription factors such as HIF-1 alpha is a promising approach since it mimics natural angiogenesis in that a plurality of growth factors are induced following transfection [26,28]. However clinical results are too premature to draw firm conclusions. Additionally, the transfection of foreign genes may possess unintended consequences in the long run due to the uncontrolled nature of the transfected gene insert.

## Animal studies using stem cell therapy for CLI

Cellular therapy for CLI is based on the rationale that delivery of endothelial progenitor cells into areas of ischemia may result not only in differentiation of the cells into endothelium and thus contribute to angiogenesis, but also produce various growth factors capable of stimulating angiogenesis through paracrine interactions with adjacent cells. One of the first observations that bone marrow contains high numbers of endothelial progenitor cells was by Ashara et al using infusion of bone marrow cells in which the beta-galactosidase marker was expressed under control of endothelial cell-specific promoters such as Flk-1 and Tie-2. EPC derived from the genetically marked bone marrow were shown to promote physiological (corpus luteal and wound healing), and pathogenic (cancer) angiogenesis. The same study demonstrated that administration of bone marrow cells into muscles of mice with induced hindlimb ischemia led to neoangiogenesis mediated in part by the transferred cells [29].

A more extensive investigation of bone marrow administration of treatment of a model of CLI utilized the rat femoral ligation model and assessed not only perfusion but also the functional endpoint of treadmill running. Administration of syngeneic bone marrow in six locations proximal to the area of ligation caused a significant increase in perfusion, as well as treadmill performance in the treated rats [30]. A study using a similar model demonstrated not only improved perfusion subsequent to bone marrow administration but also enhanced production of the angiogenic cytokines IL-1 and FGF-2 [31]. Since diabetes is associated with CLI, one question addressed in animal experiments is whether the diabetic state influences angiogenic ability of autologous bone marrow cells. Using the femoral artery ligation model, it was demonstrated that cellular therapy was able to successfully induce angiogenesis in streptozoicin treated diabetic rats [32]. Subsequent to these experiments, numerous variations on a theme have been performed, all illustrating the ability of bone marrow and other cellular populations to induce therapeutic angiogenesis in animal models. For example, implantation of whole bone marrow was effective at inducing formation of collateralization in a model of CLI involving double ligation of the left femoral artery just distal to the profunda femoral artery branch. Angiogenesis was demonstrated by histology and presence of endothelial cells possessing donor-specific marker [33]. Cumulatively, animal studies demonstrated that stem cells of a wide variety of sources are capable of both generating angiogenic cytokines that promote endogenous stem cells and reparative processes, and directly promote angiogenesis via EPCs. The next question was whether this could be translated into clinical applications.

## Clinical use of stem cell therapy for CLI

Bone marrow transplantation has been clinically used for hematopoietic reconstitution for more than 40 years, thus procedures for bone marrow extraction and manipulation are routine. In 2002 Tateishi-Yuyama et al [34] used the technique of bone marrow extraction and mononuclear cell preparation to generate an autologous source of stem cells for CLI treatment. In total 45 patients were injected with mononuclear cells at concentrations ranging between 0.7–2.8 × 10^9 ^cells in the gastrocnemius muscle of the ischemic leg using approximately 40 injections in a volumes of 0.75 ml. Statistically significant increases in ankle brachial index, trancutaneous oxygen pressure, pain free walking time, and amelioration of rest pain were detected at 4 and 24-week follow-up. The investigators also demonstrated production of proangiogenic cytokines by the injected cells. Other groups also reported similar therapeutic results: Nizankowski et al treated 10 CLI patients with condensed bone marrow stem cells and observed improvement using laser doppler flux and percutaneous oxygen partial pressure, as well as decrease in pain severity. Interestingly no correlation was found between cell number injected and effect [35]. Durdu et al performed intramuscular injection of autologous bone marrow mononuclear cells to 28 CLI patients who were nonresponders to conservative therapy and ineligible for revascularization. Of the 28 patients, only 1 needed amputation. Statistically significant increases in rest pain scores, peak walking time, and quality of life were noted. Angiography evidence of collateral vessel formation was observed in 22 of the patients at 6 months [36]. Numerous other studies have demonstrated safety and efficacy of autologous bone marrow stem cell therapy [37,38], as well as for autologous mobilized stem cells [39-41]. A review of the total 18 clinical studies published in the English language using autologous stem cell therapy for CLI overviews the above mentioned studies in detail [42].

Thus, autologous bone marrow therapy for CLI appears to be a promising solution to the current lack of treatments in patients ineligible for endovascular/surgical interventions. However, this is limited by the need for anesthesia during the bone marrow extraction procedure, which is dangerous in the CLI population since numerous co-morbidities exist [43]. Mobilization using G-CSF alleviates the need for bone marrow puncture, however use of this cytokine is feared by some to predispose CLI patients to thrombosis due to the potent leukocyte mobilization that occurs subsequent to its administration [44].

Additionally, there are suggestions in the literature that endothelial progenitors from bone marrow of patients with peripheral artery disease may be suboptimal in comparison to other progenitor sources [45,46]. Specifically, these findings suggest firstly that an age-associated decline in angiogenic potential of bone marrow cells occurs, and secondly, patients with peripheral artery disease have a significantly inhibited angiogenic potential as compared with age-matched controls. One explanation for this is that stem cell depletion occurs due to increased demand for new progenitor cells in the damaged vasculature [47]. An "off the shelf" product that is collected from young volunteers and selected for potent angiogenic activity may overcome this drawback.

## Endometrial regenerative cells

Dr Taylor's group demonstrated that various endometrial cells are actually derived from bone marrow. Through studying endometrial stromal cells isolated from female recipients of male bone marrow, they reported positive staining for the Y-chromosome in a substantial number of cells [48]. Reports of adherent, STRO-1 positive cells that can be isolated from the endometrium have been made by Dr. Taylor and other groups as well [49-51]. Given the high rate of angiogenesis associated with build up of the uterine lining, it is known that various proangiogenic cells and possibility EPC are found. We have reported a unique population of cells derived from the menstrual blood, which after cloning we termed Endometrial Regenerative Cells (ERC) [52]. We believe the ERC represents a unique population of cells in contrast to endometrial stromal cells based on: a) different rate of proliferation (ERC proliferate approximately once every 19 hours, whereas stromal cells every 24–36 hours); b) ability to differentiate into a wide number of tissues, stromal cells have only been shown to differentiate into bone, fat, and cartilage; c) ERC lack expression of STRO-1, whereas other stromal derived cells express this marker.

Cells resembling some phenotypic characteristics of ERC appear to be similar to the menstrual blood derived mesenchymals described by Miyoshi's group [53]. These cells express CD29, CD105 and are negative for CD34 and CD45. The investigators demonstrated cardiogenic potential of these cells both in vitro and in vivo, without observation of carcinogenic changes. Patel's group reported a "multipotent menstrual blood stromal stem cell" [54], which had an expression profile of Oct-4, c-kit and SSEA-4 positive and negative for expression of hematopoietic markers. Given that menstrual blood contains numerous types of cellular populations, it is possible that each of these two other groups have cells that are unique, however express certain commonalities. Regardless of differences, all three groups have demonstrated safety in terms of lack of teratogenic potential, some degree of pluripotency, and ability to be rapidly expanded in tissue culture.

## Angiogenic potential of ERC

**Figure 1:**
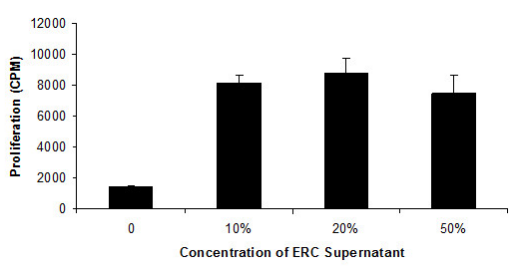
**Stimulation of HUVEC proliferation by ERC conditioned media**. ERC conditioned media was generated by 5 day culture of 10 × 10(5) cells in completed DMEM media. Conditioned media was added at the indicated concentrations to HUVEC cells in 96 well plates at a concentration of 5000 cells per well. Cells were incubated for 72 hours and proliferation as assessed by thymidine incorporation. Error bars represent ± Standard Deviation.

**Figure 2:**
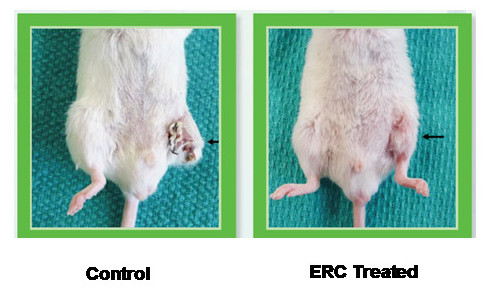
**Limb preservation by ERC administration**. 16 BALB/c female mice (6–8 weeks of age, Jackson Labs, Bar Harbor, Maine) underwent unilateral ligation of the femoral artery and its branches (superficial eplgastrlc artery) for induction of the limb ischemia. Additionally, ligation of N. peroneus for reproducing a neurotrophic ulcer-like injury was performed. Mice were divided into 2 groups of 8. Immediately after induction of injury, 1 million ERC were injected into the hind-limb muscle below the area of ligation. Cells were also injected on day 0, day 2 and day 4. ERC where injected in a volume of 200 microliters of saline. By day 14, necrosis was observed in legs of 8 control mice. 8 mice treated with ERC had intact limbs, with 2 displaying signs of impeded walking.

The endometrium undergoes rapid angiogenesis in a controlled manner monthly. Upregulated production of angiogenic factors including PDGF, EGF, and VEGF have been described both in the mouse and human endometrium [55,56]. Some degree of similarity exists between ERC and bone marrow derived mesenchymal stem cells, for example, expression of CD90, CD105 and lack of CD45 and CD34 [57]. Bone marrow derived mesenchymal stem cells are potently angiogenic [58]. Therefore we performed a series of preliminary experiments assessing proof of concept of angiogenic activity. Given that conditioned media from pro-angiogenic cells has previously been demonstrated to stimulate HUVEC proliferation [59], we used this assay to assess soluble angiogenic activity. As seen in Figure 1, a dose dependent stimulation of HUVEC proliferation was observed. This is in agreement with our previous publication demonstrating potent secretion of angiogenic cytokines such as PDGF-BB and angiopoietin [52]. In order to assess in vivo angiogenic activity, a pilot study involving 8 treated and 8 control mice was performed. All animals were cared for in accordance with the guidelines established by the Canadian Council on Animal Care. Immune competent BALB/c mice were administered approximately 1 million ERC intramuscularly after ligation of the femoral artery and its branches (superficial epigastrlc artery) for induction of the limb ischemia. Additionally, excision of *N. peroneus *for reproducing a neurotrophic ulcer-like injury was performed. Mice were divided into 2 groups of 8. Immediately after induction of injury, 1 million ERC were injected into the hind-limb muscle below the area of ligation. Cells were also injected on day 0, day 2 and day 4. ERC where injected in a volume of 200 microliters of saline. By day 14, necrosis was observed in legs of 8 control mice. 8 mice treated with ERC had intact limbs, with 2 displaying signs of impeded walking. Figure 2 depicts a representative control and treated mouse. These data, although preliminary, suggest that possibility of using ERC to stimulate angiogenesis. Perhaps most strikingly was that the therapeutic effect was observed despite utilization of human cells in an immune competent xenogeneic animal.

## Allogeneic barriers

Mesenchymal stem cell populations appear to possess active immune modulatory properties in vitro and in vivo. Specifically, bone marrow derived mesenchymal cells have been demonstrated to inhibit ongoing mixed lymphocyte reaction (MLR) [60], induce generation of T regulatory cells [61], and in vivo suppress autoimmunity such as collagen induced arthritis [62] and experimental allergic encephalomyelitis [63]. In animal models, acceleration of wound healing [64], or post-infarct recovery [65], may be accomplished by administration of allogeneic mesenchymal cells. Allogeneic cord blood derived mesenchymal stem cells have demonstrated clinical benefit in a small series of patients with CLI caused by Buerger's Disease [66]. Perhaps most convincingly, allogeneic bone marrow derived mesenchymal stem cells have passed Phase I clinical trials (safety) and Phase II (preliminary efficacy) and now are in Phase III trials for graft versus host disease and Crohn's by the company Osiris Therapeutics [67]. Thus there appears to be support for the allogeneic use of bone marrow derived mesenchymal cells for various applications. The question is whether ERC possess similar immune modulatory properties. This is particularly important since in many situations autologous ERC will either not be available or will not be practical.

**Figure 3:**
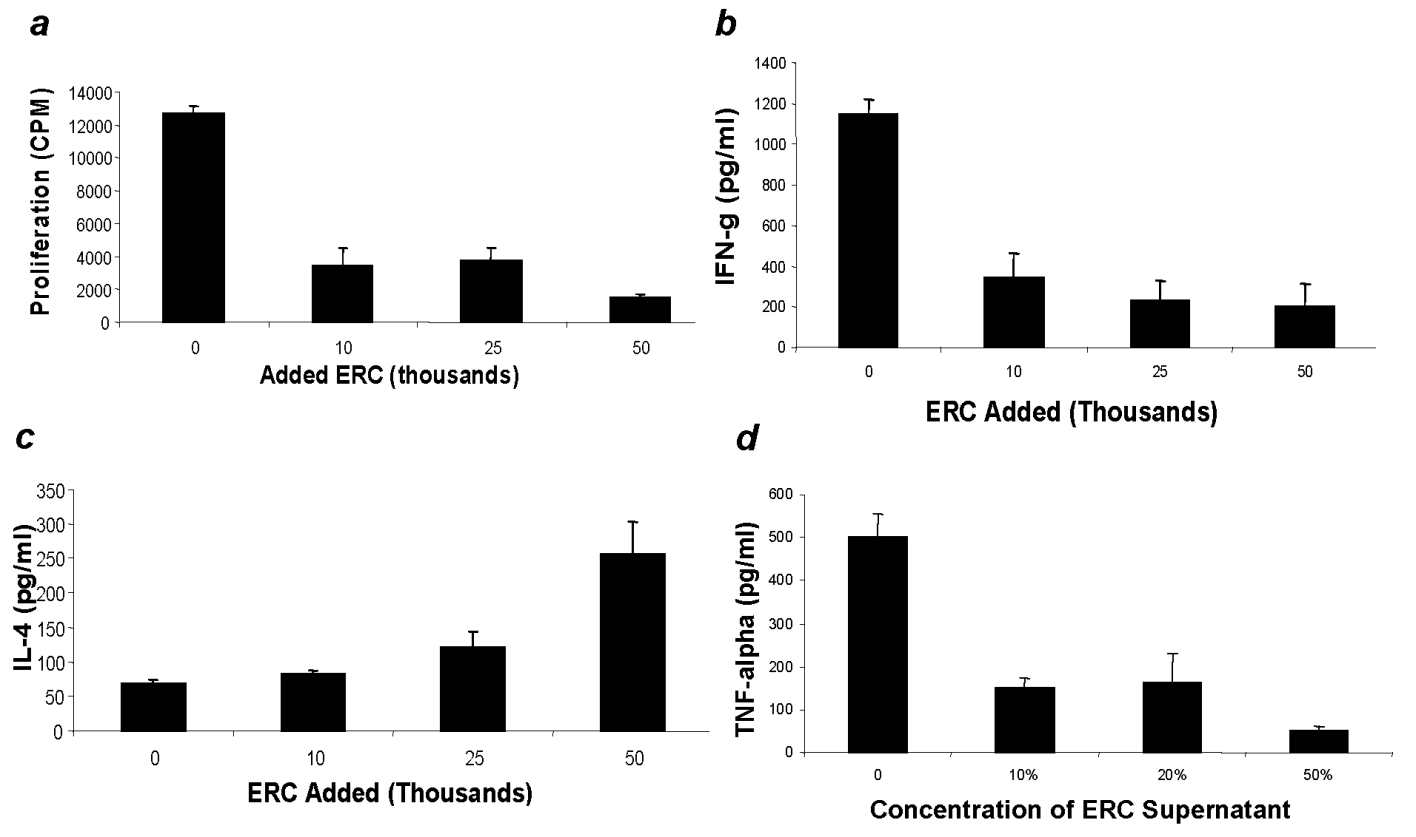
**Immune modulation by ERC**. ***A***) In order to assess active proliferation of ongoing MLR 50,000 mitotically inactivated (mitomycin C) PBMC (stimulators) were incubated with 50,000 allogeneic PBMC (responders). Mitotically inactivated ERC were added at the indicated concentrations per well. Cells were cultured for 72 hours. For the last 18 hours of culture, cells were pulsed with 0.5 μCi 3H-thymidine. ***B&C***) Ongoing MLR was established as described and supernatant was collected at48 hours. IFN-gamma and IL-4 were assessed by Quantikine Sandwich ELISA (R&D Systems, Minneapolis). ***D***) Suppression of TNF-alpha production by ERC Conditioned Media. ERC conditioned media was added to mouse splenocytes that were activated with 2.5 microliters of LPS in a total volume of 200 microliters. The concentration of splenocytes was 250,000 cells per well. After culture for 48 hours, supernatant was examined for TNF-alpha by ELISA (R&D Systems). Error bars represent ± Standard Deviation.

In preliminary experiments we have demonstrated that addition of ERC to ongoing mixed lymphocyte reactions results in suppression of proliferation (Figure 3a), inhibition of IFN-gamma (Figure 3b), stimulation of IL-4 (figure 3c) and inhibition of TNF-alpha after LPS stimulation (Figure 3d). These results support the notion that ERC may share immune modulatory properties with bone marrow derived mesenchymal stem cells. Back to back comparisons between ERC and bone marrow derived MSC have demonstrated equivalent, or in some cases, superior immunomodulatory potential based on inhibition of mixed lymphocyte reaction.

## Clinical entry of ERC

Clinical entry of novel cellular therapies requires demonstration of safety and reasonable possibility of improving the patient's condition. Previous studies involving long term follow up of animals treated with ERC, as well as karyotypic normality of these cells after extended passage (68 doublings) have demonstrated lack of tumorogenicity [52]. In fact, administration of ERC into 18 SKH1 treated with 2240 J/m2 UVB radiation three times a week for 10 weeks to induce skin tumors did not accelerate tumor formation. Based on what appears to be a favorable safety profile, as well as preliminary supportive data, we propose a dose escalating clinical trial evaluating intramuscular administration of ERC in patients with CLI ineligible for surgical or endovascular intervention. Given several biological similarities between bone marrow derived mesenchymal stem cells and ERC, as well as side-by-side growth factor production data, dose escalation may be performed from 10, 25, and 50 million cells per limb. One unique aspect of using ERC for this indication is the possibility of administering allogeneic, standardized cell populations. In addition to classical endpoints of success in CLI trials such as imaging for collateral vessel formation, pain free walking, ABI, and Fountain Score, ERC trials should include serum cytokine production and evaluation of endogenous stem cell mobilization. In conclusion, the ERC represents a novel approach the CLI by offering an "off the shelf" therapeutic that circumvents pitfalls of other approaches currently in use. The proposed clinical trial will shed light of the viability of this hypothesis.

## Competing interests

Neil Riordan and Thomas Ichim are shareholders and management of Medistem Inc (OTCBB:MEDS).

## Authors' contributions

All authors read and approved the final manuscript. MP, HW, AP, SK, NA, KC, AM, AP, EC, TI, and NR concieved experiments, interpreted data, and wrote the manuscript.
